# Identifying Prognostic Biomarkers Related to m6A Modification and Immune Infiltration in Renal Cell Carcinoma

**DOI:** 10.3390/genes13112059

**Published:** 2022-11-07

**Authors:** Junjie Ye, Peng Li, Huijiang Zhang, Qi Wu, Dongrong Yang

**Affiliations:** 1 The Second Affiliated Hospital of Soochow University, Suzhou 215000, China; 2 Lishui City People’s Hospital, Lishui 323000, China

**Keywords:** renal cell carcinoma, biomarker, immune infiltration, m6A modification

## Abstract

Background: Renal cell carcinoma (RCC) is the largest category of kidney tumors and usually does not have a good prognosis. N6-methyladenosine(m6A) and immune infiltration have received increased attention because of their great influence on the clinical outcome and prognosis of cancer patients. Methods: We identified hub genes through multi-dimensional screening, including DEGs, PPI analysis, LASSO regression, and random forest. Meanwhile, GO/KEGG enrichment, cMAP analysis, prognostic analysis, m6A prediction, and immune infiltration analysis were performed to understand the potential mechanism and screen therapeutic drugs. Results: We screened 275 downregulated and 185 upregulated genes using three GEO datasets and the TCGA dataset. In total, 82 candidate hub genes were selected using STRING and Cytoscape. Enrichment analysis illustrated that the top 3 biological process terms and top 1 KEGG term were related to immunity. cMAP analysis showed some antagonistic molecules can be candidate drugs for the treatment of RCC. Then, six hub genes (ERBB2, CASR, P2RY8, CAT, PLAUR, and TIMP1) with strong predictive values for prognosis and clinicopathological features were selected. Meanwhile, P2RY8, ERBB2, CAT, and TIMP1 may obtain m6A modification by binding METTL3 or METTL14. On the other hand, differential expression of CAT, ERBB2, P2RY8, PLAUR, and TIMP1 affects the infiltration of the majority of immune cells. Conclusions: We identified six hub genes through multi-dimensional screening. They all possess strong predictive value for prognosis and clinicopathological features. Meanwhile, hub genes may regulate the progression of RCC via an m6A- and immunity-dependent mechanism.

## 1. Introduction

Renal cell carcinoma (RCC) is a common subtype of kidney tumor, with approximately 73,000 new cases and 14,000 deaths each year [1]. Considering its association with an unhealthy lifestyle, genetic alteration, and cell injury, RCC is a complex, multifactorial illness. Despite huge developments in diagnosis, surgery, and medication, RCC still has an unsatisfactory outcome [2]. The prognosis is poor, especially for advanced RCC patients whose 5-year survival rate is less than 5% because of insensitivity to chemotherapy and other treatments [3]. Therefore, more efficient targets for diagnosis and treatment are urgently needed.

m6A (N6-methyladenosine) is the most common and reversible RNA modification in eukaryotic cells. It is a dynamic modification regulated by methyltransferases, demethylases, and RNA-binding proteins [4]. Increasing evidence has revealed that m6A modification is associated with several biological processes, such as cell proliferation, apoptosis, tumor progression, damage repair, and immunomodulatory disorders [5]. m6A-related enzymes can alter the m6A levels of cancer inhibitors or promoters affecting tumor progression, such as BRD4, MYC, SOCS2, and EGFR [6].

On the other hand, the tumor immune microenvironment (TIME) has received much attention because of its great influence on the clinical outcome and prognosis of cancer patients [7]. Thus, the infiltration of immune cells is considered to be an appealing target for diagnosis and treatment [8]. Meanwhile, some genes can promote or inhibit the infiltration of immune cells. For example, the TBC1D3 family can function as a prognostic biomarker of RCC, and the expression of TBC1D3 has a negative correlation with the infiltrated level of CD4+ T cells [9]. Additionally, TREM2 is regarded as a prognostic marker in several cancers, and the expression of TREM2 in these tumors is positively related to the infiltration level of most immune cells [10].

In this study, we explored six promising biomarkers that have a good correlation with the survival prognosis and clinicopathological features of RCC. We also analyzed their relevance to immune infiltration and m6A modification. Moreover, we presented a preliminary landscape of immune cell infiltration and m6A-related genes in RCC.

## 2. Materials and Methods

### 2.1. Data Collection

Transcription profile data were downloaded from the GEO database with the following criteria. First, the dataset should contain patients and controls. Second, patients and controls of each group should include more than 3 tissue samples from homo sapiens. Third, the data type is expression profiling by array. RNA-seq data of ccRCC and related clinical information were downloaded from the TCGA website. In this study, three GEO datasets and the TCGA dataset were applied to DEG (differentially expressed gene) analysis: GSE168845 (4 normal samples and 4 cancer samples https://www.ncbi.nlm.nih.gov/geo/query/acc.cgi?acc=GSE168845, accessed on 20 March 2022), GSE71963 (16 normal samples and 32 cancer samples https://www.ncbi.nlm.nih.gov/geo/query/acc.cgi?acc=GSE71963, accessed on 20 March 2022), GSE68417 (19 normal samples and 29 cancer samples https://www.ncbi.nlm.nih.gov/geo/query/acc.cgi?acc=GSE68417, accessed on 20 March 2022), and TCGA dataset (72 normal samples 539 cancer samples https://portal.gdc.cancer.gov/projects/TCGA-KIRC, accessed on 20 March 2022).

### 2.2. DEG Identification

The DEGs between RCC and normal tissues were identified using the R package “limma” with the threshold set at FoldChange > 2 and adjusted *p* < 0.05. The results were visualized by the R package “ggplot2”. Then, the intersection of the three GEO datasets and the TCGA dataset was calculated and displayed in the form of a Venn diagram.

### 2.3. PPI (Protein–Protein Interaction) Network Construction and Candidate Hub Gene Identification

The PPI network, predicting the functional interactions between genes, could provide insights into the molecular mechanism. The STRING online database (http://string-db.org accessed on 26 September 2022) [11] was utilized to construct the PPI network. Cytoscape is a useful software capable of visualizing molecular interaction networks [12]. MCODE, a plugin of Cytoscape, was used to extract the densely connected subnetwork with the threshold set at MCODE default parameters (node score cutoff ≥ 0.2, degree cutoff ≥ 2, K-core ≥ 2, and max depth = 100). CytoHubb, another plugin of Cytoscape, was applied to identify the top 100 proteins ranked by the MCC algorithm. Then, the intersected molecules were recognized as the candidate hub genes.

### 2.4. GO Enrichment and KEGG Pathway Analysis of Candidate Hub Genes

GO enrichment is a useful bioinformatics method to analyze biological functions, including biological processes, molecular functions, and cellular components. KEGG pathway analysis is also widely utilized to provide functional annotations for a gene set including biological pathways, drugs and chemical substances, and diseases. In this study, we performed GO and KEGG enrichment analyses by the R packages “ggplot2” and “clusterprofiler” with the threshold set at adjusted *p* < 0.05.

### 2.5. cMAP Analysis of Candidate Hub Genes

The cMap database is the largest perturbation-driven gene expression dataset and is a resource using cellular responses to perturbation to find relationships between diseases, genes, and therapeutics (https://clue.io/, accessed on 26 September 2022) [13]. In this study, antagonistic molecules were screened for RCC by cMAP analysis of 82 candidate hub genes. The query parameters selected were “Gene expression (L1000)” and “Latest”. The top 10 ranked molecules and relevant information are listed in Table 1.

### 2.6. Hub Gene Identification

LASSO is a frequently used algorithm to screen the most valuable genes from high-dimensional data. Random forest is a supervised learning method. An important property of a Random forest is that it can give an importance measure for every feature. So it was widely used for the extraction of key features from a large gene set [14]. In our research, LASSO and random forest were performed together to select the most powerful variables for survival prognosis. LASSO was performed by “glmnet” package in R. The lambda (λ) value of LASSO was set at lambda.min after ten rounds of cross-validation. Random forest was performed by “randomForestSRC” package in R. “ntree” set at 1000, “nsplit” set at 1, “method” set at “md”.

### 2.7. Survival and Clinicopathological Characteristic Analysis of Hub Genes

The overall survival and disease-free survival analyses of the hub genes were performed using the GEPIA website (http://gepia.cancer-pku.cn/, accessed on 26 September 2022) [15]. The correlation analysis between the expression of hub genes and clinicopathological features was carried out by UALCAN (http://ualcan.path.uab.edu/, accessed on 26 September 2022) [16].

### 2.8. Correlation between Hub Genes and m6A-Related Genes

Considering that m6A-related genes are discovered continuously, some of them may not be included in our research. We selected multiple representative genes after reading the relevant literature, such as the methyltransferases METTL14, METTL3, WTAP, KIAA1429, ZC3H13, and RBM15, demethylases ALKBH5 and FTO, and RNA-binding proteins YTHDC1/2, YTHDF1/2/3, HNRNPA2B1, FMR1, and LRPPRC [17]. The expression of the m6A regulators in tumor and normal tissues was analyzed using the GEPIA database (http://gepia.cancer-pku.cn/index.html, accessed on 26 September 2022) [15]. Correlation analysis between hub genes and m6A-related genes was performed by TIMER2.0 (http://timer.cistrome.org/, accessed on 26 September 2022) [18]. Finally, the possible m6A sites of the hub genes were predicted using SRAMP (http://www.cuilab.cn/sramp/, accessed on 26 September 2022) [19]. The predicted interaction plot was shown in a sankey using the data from ENCORI web (https://starbase.sysu.edu.cn/, accessed on 26 September 2022) and the catPARID database (http://www.tartaglialab.com/, accessed on 26 September 2022) [20]. First, we queried the interactive possibility of 16 m6A-related genes with hub genes in ENCORI. Then, the interactive score was confirmed and obtained in the catPARID database.

### 2.9. Immune Infiltration Analysis

CIBERSORTX is a powerful method for characterizing cell subsets by gene expression profiles [21]. In our study, the CIBERSORTx website (https://cibersortx.stanford.edu/, accessed on 26 September 2022) was used to estimate the abundances of 22 immune cells between cancer tissue and normal tissue, high- and low-expression groups of hub genes, and different clinicopathological feature groups (t1–t2 vs. t3–t4, n0 vs. n1–n3, m0 vs. m1, grade1–grade2 vs. grade3–grade4). Gene expression profiles and Reference gene expression were needed to input into the website. Gene expression profiles came from the ccRCC and control cohort of TCGA. Reference gene expression signatures were LM22 (22 immune cell types) provided by the website. The permutation for significance analysis chose 100. The median value of hub genes was defined as the threshold of high and low expression. Then, R packages (“ggplot2”, “vioplot”, “corrplot”, “survial”) were used to visualize the results. *p* < 0.05 was considered to be significantly different.

## 3. Results

### 3.1. DEG Identification in RCC

**Figure 1:**
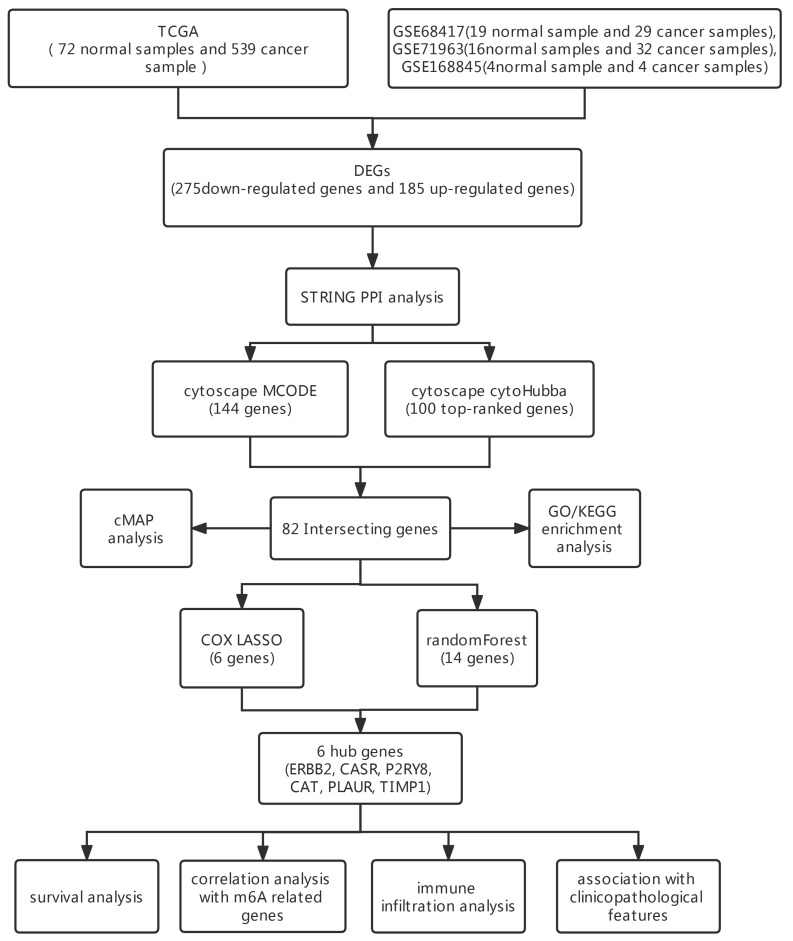
Schematic sketch of the research design. Three GEO datasets and ccRCC cohort of TCGA were included in present study. Through differential gene analysis, PPI, LASSO regression, and random-forest analysis, 6 hub genes were identified. Then, survival analysis, correlation analysis with m6A-related genes, immune infiltration analysis, and association with clinicopathological features were performed to understand 6 hub genes further.

**Figure 2:**
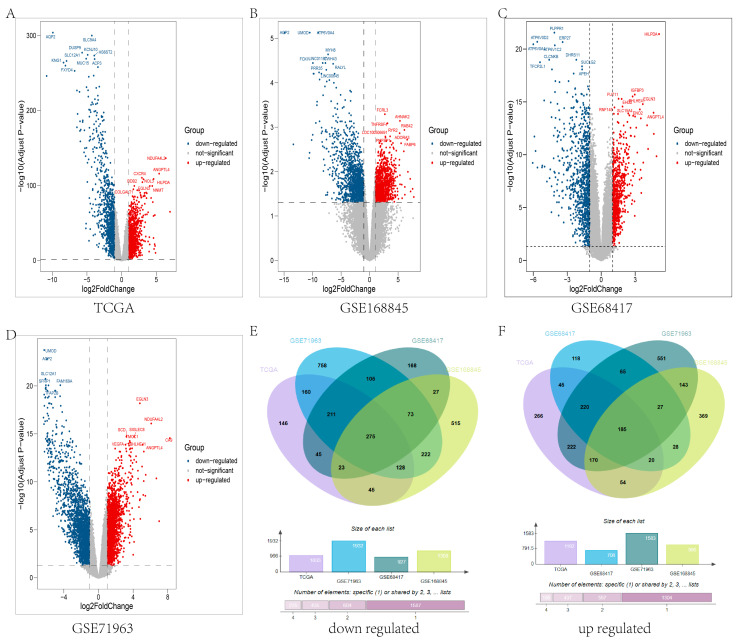
Volcano plots and Venn diagrams visualize the DEGs. (**A**–**D**) The volcano plots reveal the DEGs between RCC and normal tissues of the TCGA dataset (**A**), GSE168845 (**B**), GSE68417 dataset (**C**), and GSE71963 dataset (**D**) with thresholds set at 2-fold change and *p* < 0.05. Blue points indicate downregulated genes, and red points represent upregulated genes. (**E**) Venn diagram displaying the intersection of downregulated DEGs. (**F**) Venn diagram displaying the intersection of upregulated DEGs. The bars in (**E**,**F**) mean the size of DEG lists of TCGA, GSE71963, GSE68417, and GSE168845.

The workflow of this research is shown in Figure 1. There were 708, 2037, 996, and 1183 upregulated genes and 927, 2442, 1308, and 1034 downregulated genes in the GSE68417, GSE71963, GSE168845, and TCGA datasets, respectively. The DEGs of the three GEO datasets and TCGA dataset are illustrated by volcano maps (Figure 2A–D). The intersection of 185 upregulated genes and 275 downregulated genes between the 4 DEG lists is shown by a Venn diagram (Figure 2E,F).

### 3.2. PPI Interaction Network and Identification of Candidate Hub Genes

**Figure 3:**
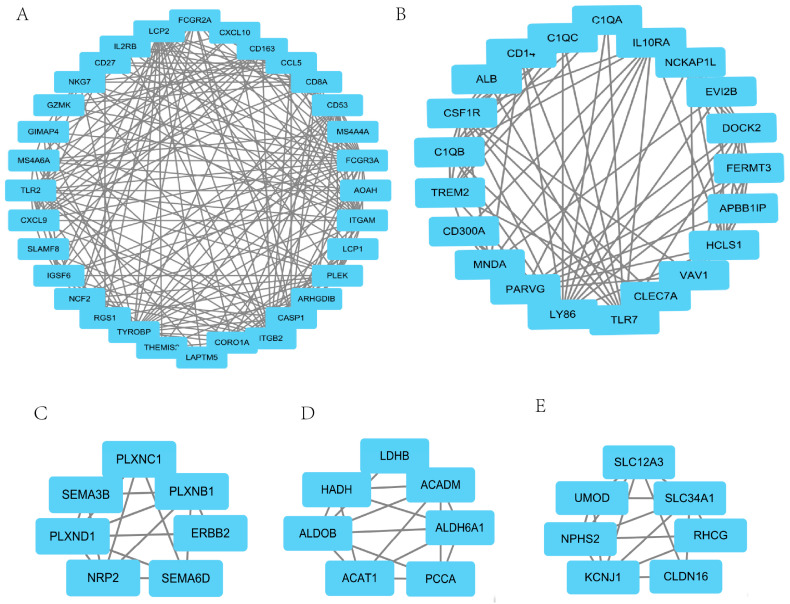
The PPI subnetwork with an MCODE score > 5.0. PPI subnetworks were extracted by the plugin MCODE. The clusters with MCODE scores > 5.0 are displayed. (**A**) The largest cluster with 32 genes, MCODE score 13.742. (**B**) The cluster with 21 genes, MCODE score 8.7. (**C**) The cluster with 7 genes, MCODE score 5.333. (**D**) The cluster with 7 genes, MCODE score 5.333. (**E**) The cluster with 7 genes, MCODE score 5.333.

STRING and Cytoscape were applied together to identify candidate hub genes. Fourteen clusters with 144 genes (gene list 1) were selected using the plugin MCODE of Cytoscape. The MCODE scores ranged from 3.0 to 13.742. The largest cluster contains 32 genes with an MCODE score of 13.74. The smallest cluster has 3 genes with an MCODE score of 3.0. The clusters with MCODE scores > 5.0 are shown in Figure 3. The top 100 genes (gene list 2) ranked by the MCC algorithm were selected using the plugin cytoHubb of Cytoscape. Eighty-two genes overlapping in gene list 1 and gene list 2 were identified as candidate hub genes. Gene list1, gene list2, and candidate hub gene list were displayed in Supplementary Table S1.

### 3.3. GO and KEGG Enrichment Analysis

**Figure 4:**
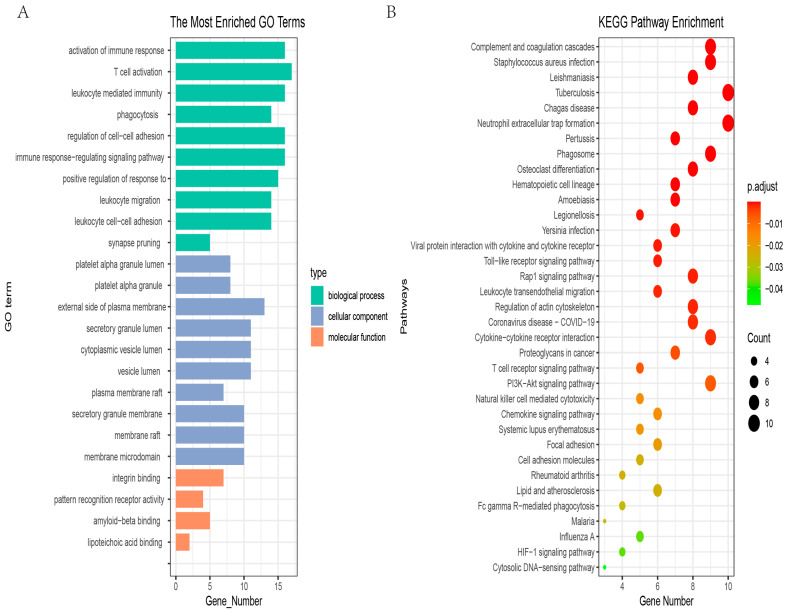
Gene Ontology (GO) and KEGG enrichment analyses of 82 candidate hub genes. (**A**) The top enriched terms of biological process, cellular component, and molecular function; (**B**) KEGG pathway mainly enriched in complement and coagulation cascades, Staphylococcus aureus infection, leishmaniasis, and tuberculosis.

To understand the potential biological functions of DEGs, GO and KEGG enrichment analyses were performed by the R package “clusterprofiler”. In total, 35 KEGG pathways, 4 molecular function terms, the top 10 biological process terms, and the top 10 cellular component terms are shown in Figure 4A,B. The molecular function terms included integrin binding, pattern recognition receptor activity, amyloid−beta binding, and lipoteichoic acid binding. The biological process terms were mainly enriched in lipoteichoic acid binding, T-cell activation, and phagocytosis. The cellular component terms mainly included synapse pruning, platelet alpha granule lumen, and platelet alpha granule lumen. The enriched KEGG pathways were mainly involved in complement and coagulation cascades, Staphylococcus aureus infection, leishmaniasis, and tuberculosis. Meanwhile, the top 3 biological process terms and the top 1 KEGG term were related to immunity. This enrichment result verifies previous research results that immunity is one of the major mechanisms of candidate hub genes [22].

### 3.4. cMAP Analysis of Candidate Hub Genes

After cMAP analysis of 82 candidate hub genes, we found their expression treated by some molecular drugs was basically contrary to that induced by ccRCC. Thus, they can be candidate drugs for the treatment of RCC. The top 10 molecules ranked by the raw score are shown in Table 1. For example, the top molecule is miglustat. It can target UGCG, GAA, and SLC5A4 and act as a glycosylation inhibitor.

### 3.5. Hub Gene Identification

**Figure 5:**
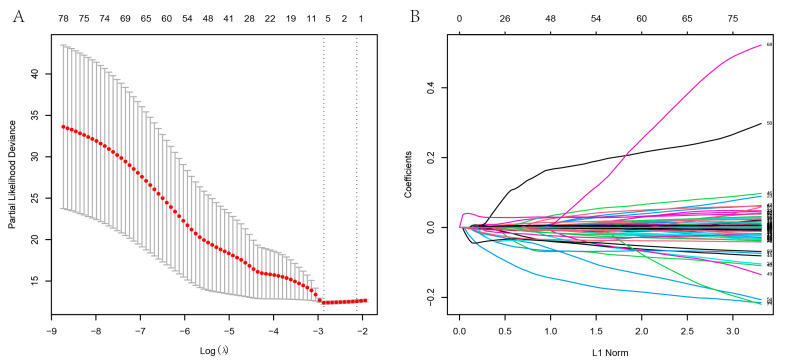
LASSO regression of candidate hub genes. (**A**) The solid lines were the partial likelihood of deviance. The dotted vertical lines drawn on the right indicated the optimal values of log(λ) through 10-fold cross-validation. (**B**) LASSO coefficient profiles of candidate hub genes. Each curve represented a gene. The curve displayed the path of gene’s coefficient against the L1-Norm. The number of nonzero coefficients at the current was defined as the effective degrees of freedom (df) for the LASSO.

Eighty-two candidate hub genes are too many to guide clinical work. Therefore, we further screened variables using the RandomForest algorithm and LASSO regression. Eighteen genes (gene list3) were selected by the random forest method, and six genes (gene list4) were selected using LASSO regression. The LASSO screening process is visualized in Figure 5. Finally, we integrated the results of LASSO regression and RandomForest to identify six hub genes: ERBB2, CASR, P2RY8, CAT, PLAUR, and TIMP1. Gene list3 and gene list4 were displayed in Supplementary Table S1. To understand the characterized functions of six hub genes, a preliminary summary performed using Genecard (https://www.genecards.org/, accessed on 26 September 2022) was supplied in Supplementary Table S2.

### 3.6. Survival and Clinicopathological Characteristic Analysis of Hub Genes

**Figure 6:**
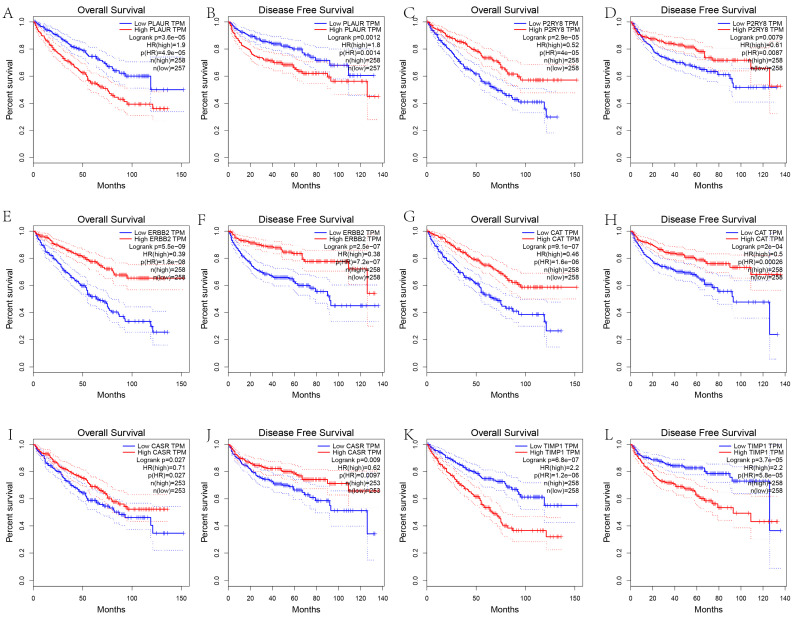
The overall survival and disease-free survival analysis of six hub genes using GEPIA. (**A**,**C**,**E**,**G**,**I**,**K**) The K–M curves of OS for RCC patients with high and low expression of PLAUR ((**A**), HR = 1.9, *p* < 0.001), P2RY8 ((**C**), HR = 0.52, *p* < 0.001), ERBB2 ((**E**), HR = 0.39, *p* < 0.001), CAT ((**G**), HR = 0.46, *p* < 0.001), CASR ((**I**), HR = 0.71, *p* = 0.027), and TIMP1 ((**K**), HR = 2.2, *p* < 0.001). (**B**,**D**,**F**,**H**,**J**,**L**) The K–M curves of DFS for RCC patients with high and low expression of PLAUR ((**B**), HR = 1.8, *p* = 0.0012), P2RY8 ((**D**), HR = 0.61, *p* = 0.0079), ERBB2 ((**F**), HR = 0.38, *p* < 0.001), CAT ((**H**), HR = 0.5, *p* < 0.001), CASR ((**J**), HR = 0.62, *p* = 0.009), and TIMP1 ((**L**), HR = 2.2, *p* < 0.001).

**Figure 7:**
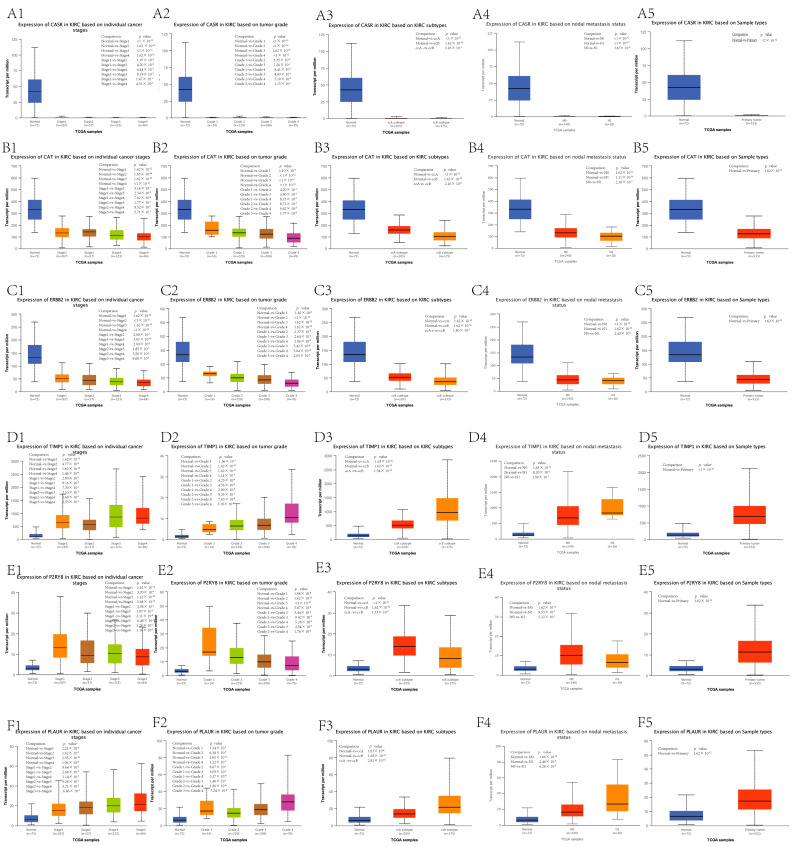
The correlation between the expression of hub genes and clinicopathological features. (**A1**–**A5**) Box diagrams visualize the expression of CASR in different clinical stages (**A1**), pathological grades (**A2**), subtypes (**A3**), numbers of metastatic lymph nodes (**A4**), and tissues (**A5**). (**B1**–**B5**) Box diagrams visualize the expression of CAT in different clinical stages (**B1**), pathological grades (**B2**), subtypes (**B3**), numbers of metastatic lymph nodes (**B4**), and tissues (**B5**). (**C1**–**C5**) Box diagrams visualize the expression of ERBB2 in different clinical stages (**C1**), pathological grades (**C2**), subtypes (**C3**), numbers of metastatic lymph nodes (**C4**), and tissues (**C5**). (**D1**–**D5**) Box diagrams visualize the expression of TIMP1 in different clinical stages (**D1**), pathological grades (**D2**), subtypes (**D3**), numbers of metastatic lymph nodes (**D4**), and tissues (**D5**). (**E1**–**E5**) Box diagrams visualize the expression of P2RY8 in different clinical stages (**E1**), pathological grades (**E2**), subtypes (**E3**), numbers of metastatic lymph nodes (**E4**), and tissues (**E5**). (**F1**–**F5**) Box diagrams visualize the expression of PLAUR in different clinical stages (**F1**), pathological grades (**F2**), subtypes (**F3**), numbers of metastatic lymph nodes (**F4**), and tissues (**F5**). *p* < 0.05 indicates that the difference is statistically significant.

Overall survival and disease-free survival analyses were conducted using the GEPIA web (Figure 6). The results demonstrated that low expression of TIMP1 and PLAUR and high expression of CASR, CAT, ERBB2, and P2RY8 were associated with positive overall survival and disease-free survival rates. Clinicopathological characteristic analysis of the hub genes was carried out by the ULUCAN web (Figure 7). CASR was highly expressed in normal tissues and expressed at extremely low levels in each clinical subgroup in cancer tissues (Figure 7A). TIMP1 and PLAUR were expressed at low levels in normal tissues. With increasing clinical stage, histological grade, and the number of metastatic lymph nodes, the expression increased (Figure 7D,F). CAT and ERBB2 were highly expressed in normal tissues. With increasing clinical stage, histological grade, and the number of metastatic lymph nodes, the expression decreased (Figure 7B,C). P2RY8 was expressed at low levels in normal tissues. However, we were surprised to find that with increasing clinical stage, histological grade, and the number of metastatic lymph nodes, the expression decreased (Figure 7E).

### 3.7. Immune Infiltration Analysis

**Figure 8:**
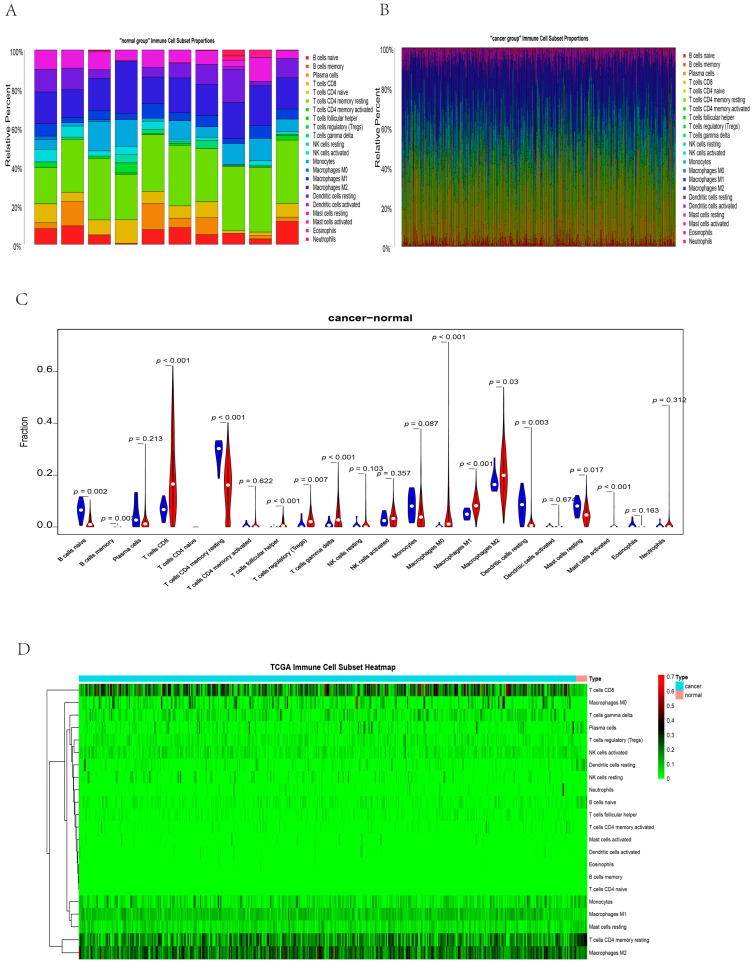
The difference in immune infiltration between RCC and normal tissues. (**A**,**B**) Stacked bar plots show the infiltration abundance of 22 immune cells in normal tissues (**A**) and RCC tissues (**B**); each bar represents one sample; macrophages and T cells account for the majority. (**C**) The violin plot illustrates the distribution of immune cells in RCC tissues and normal tissues. A large proportion of M1 macrophages, M2 macrophages, CD8 T cells, and resting memory CD4 T cells were observed in the tumor group. Resting memory CD4 T cells, M2 macrophages, monocytes, and resting dendritic cells accounted for a large part of normal tissue. B naive cells, resting memory CD4 T cells, resting dendritic cells, and resting mast cells showed greater infiltration in normal samples. (**D**) Hierarchical cluster heatmap for immune infiltration between RCC and normal tissues, each bar represents one sample.

To further determine the effect of immune infiltration in RCC and the relationship with hub genes, we performed a further analysis in three steps. First, we aimed to analyze immune infiltration in RCC and normal tissue. Stacked bar charts exhibit the proportions of 22 kinds of immune cells in normal tissue (Figure 8A) and cancer tissue (Figure 8B). A large proportion of M1 macrophages, M2 macrophages, CD8 T cells, and resting memory CD4 T cells were observed in the tumor group. Resting memory CD4 T cells, M2 macrophages, monocytes, and resting dendritic cells accounted for a large proportion of cells in normal tissue. B naive cells, resting memory CD4 T cells, resting dendritic cells, and resting mast cells were more highly infiltrated in normal samples (Figure 8C). CD8 T cells, follicular helper T cells, regulatory T cells, gamma delta T cells, M0 macrophages, M1 macrophages, M2 macrophages, and activated mast cells showed decreased infiltration in tumor tissues (Figure 8C). A hierarchical cluster plot also showed the state of immune cell infiltration in the tumor and normal groups (Figure 8D).

**Figure 9:**
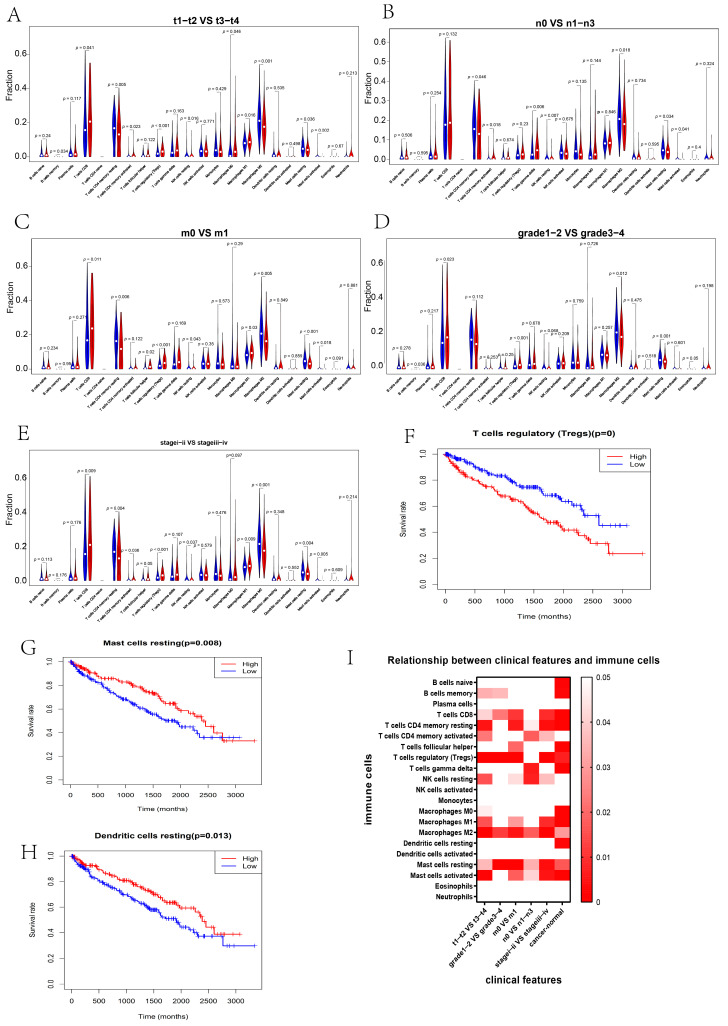
The correlation between immune infiltration and clinicopathological features. (**A**–**E**) Violin box plots display the immune infiltration of 22 immune cells between t1–t2 and t3–t4 (**A**), n0 and n1–n3 (**B**), m0 and m1 (**C**), grade 1–2 and grade 3–4 (**D**), and stage I–II and stage III–IV (**E**). (**I**) The heatmap of the *p* value depicts the difference between clinicopathological features and immune infiltrations of 22 immune cells. Red indicates that the difference in immune infiltration is significant. The more significant the difference, the darker the color. (**F**–**H**) The K–M curves of OS for high and low immune infiltration of regulatory T cells ((**F**), *p* < 0.00001), resting mast cells ((**G**), *p* = 0.008), and resting dendritic cells ((**H**), *p* = 0.013).

Second, the immune infiltration between different groups of clinicopathological features was compared (Figure 9A–E). Meanwhile, *p* values were shown together in a heatmap, and red indicated that the immune infiltration was significantly different. The more significant the difference, the darker the color (Figure 9I). Resting mast cells, regulatory T cells, and M2 macrophages had a greatly different infiltration between different situations of the primary-tumor stage, pathological grade, tumor metastasis, and clinical stage (Figure 9I). T-cell regulation was a positive predictor for the prognosis of RCC (Figure 9F). Resting mast cells and resting dendritic cells indicated a poor prognosis (Figure 9G,H).

**Figure 10:**
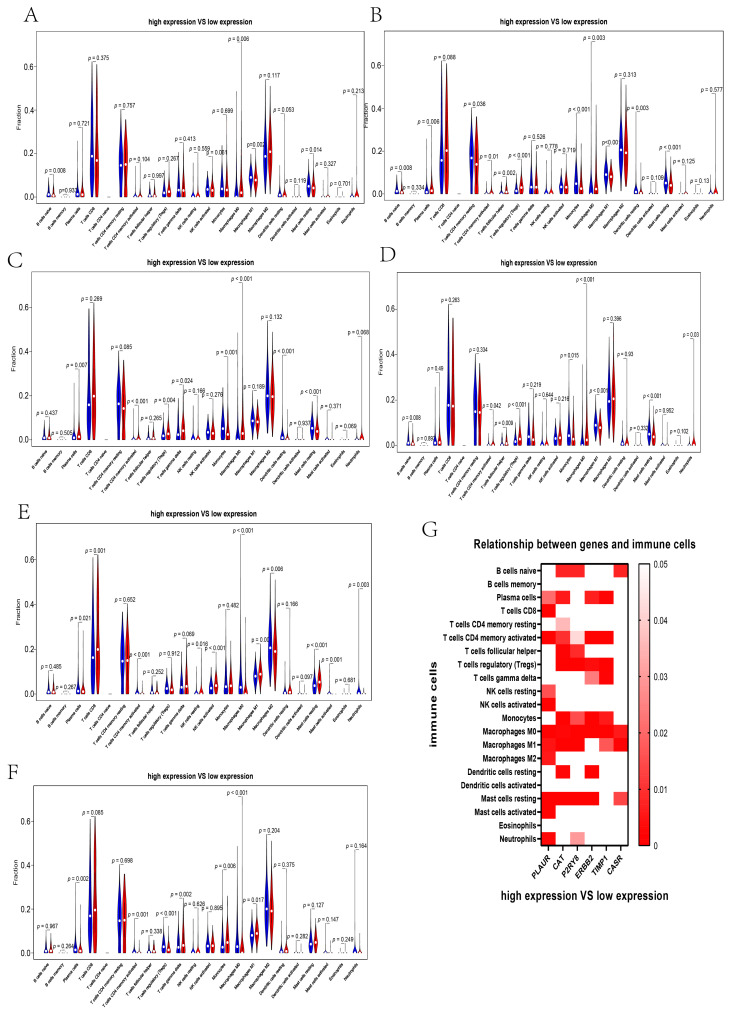
The correlation between immune infiltration and the expression of six hub genes. (**A**–**F**) Immune infiltration analysis of 22 immune cells with high and low expression of CASR (**A**), CAT (**B**), ERBB2 (**C**), P2RY8 (**D**), PLAUR (**E**), and TIMP1 (**F**). (**G**) The heatmap of the *p* value for the difference between high and low expression of hub genes and immune infiltrations of 22 immune cells. Red indicates that immune infiltration was significantly different. The more significant the difference, the darker the color.

Finally, we analyzed the relationship between the expression of hub genes and the infiltration of immune cells (Figure 10). The analysis indicated that differential expression of CAT, ERBB2, P2RY8, PLAUR, and TIMP1 affected the infiltration of the majority of immune cells (Figure 10B–F). *p* values were also shown together in a heatmap (Figure 10G).

### 3.8. Correlation Analysis between Hub Genes and m6A-Related Genes

**Figure 11:**
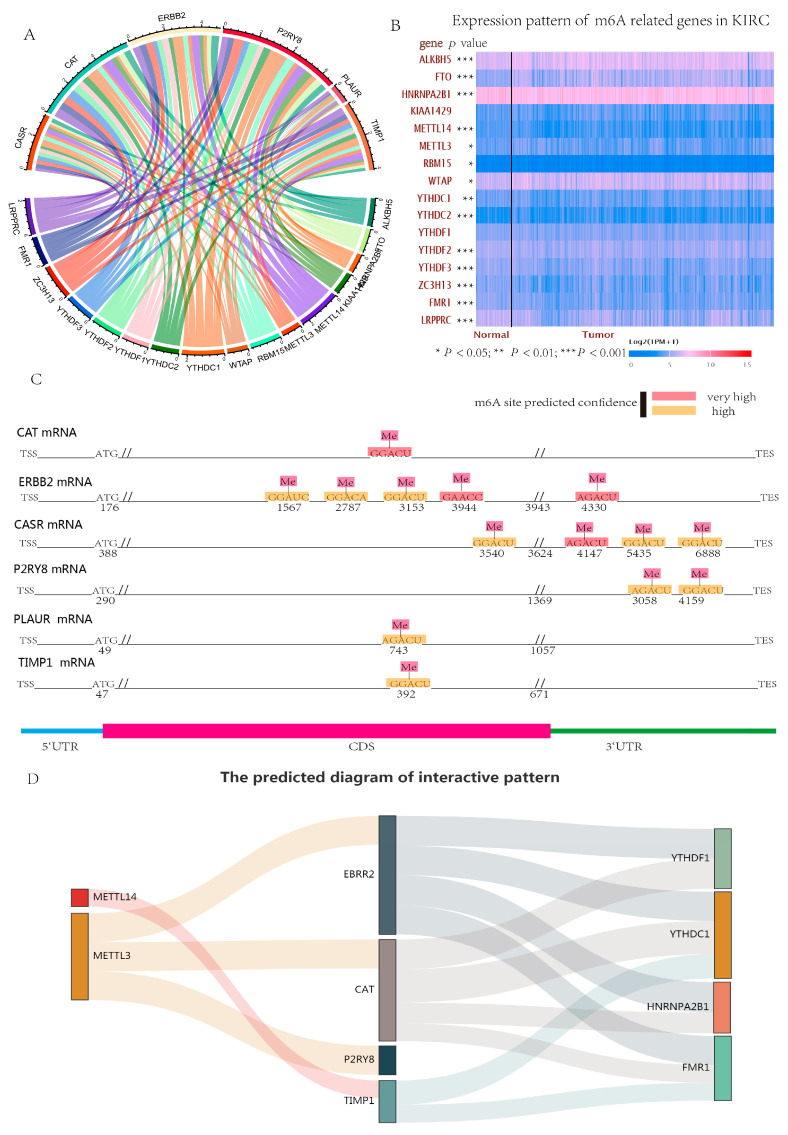
The expression of m6A-related genes, the predicted m6A sites of hub genes, and the predicted diagram of the interactive pattern. (**A**) Circos diagram visualizing the correlation strength between hub genes and m6A-related genes. The larger the gene’s region, the greater the correlation. There was a close correlation between the expression of P2RY8, ERBB2, CAT, TIMP1, and m6A-related genes. (**B**) The heatmap shows the expression of m6A-related genes between tumor and normal tissues. Among the 16 m6A-related genes, they all had differential expression, except KIAA1429 and YTHDF1. * means *p* < 0.05, ** means *p* < 0.01, *** means *p* < 0.001. (**C**) The distribution of the predicted m6A sites using the SRAMP web server based on the sequence data from Nucleotide-NCBI. Red indicates that the confidence of the predicted m6A sites is very high, and yellow indicates that the confidence is high. The numbers below the line depict the position of m6A sites. “//” represent “Start codon” and “Stop codon”. (**D**) The predicted diagram of the interactive pattern. The interactive diagram was made using data from the ENCORI and catPARID databases. First, we queried the interactive possibility of writers/erasers/readers with hub genes in ENCORI. Then, the interactive score was confirmed and obtained in the catPARID database. The thickness of the line represents the interactive possibility.

**Figure 12:**
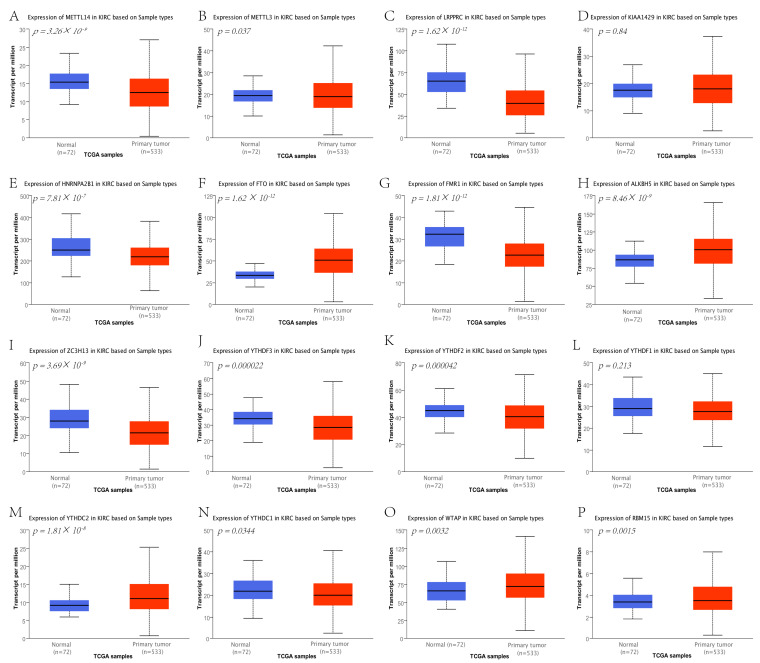
The expression of m6A-related genes using UALCAN. The box plots show the expression of m6A-related genes in RCC and normal tissues. FTO (**F**), ALKBH5 (**H**), YTHDC2 (**M**), WTAP (**O**), and RBM15 (**P**) were highly expressed in the tumor group. METTL14 (**A**), METTL3 (**B**), LRPPRC (**C**), HNRNPA2B1 (**E**), FMR1 (**F**), FMR1 (**G**), ZC3H13 (**I**), YTHDF3 (**J**), YTHDF2 (**K**), YTHDC1 (**N**) have higher expression in normal tissues. The expression of KIAA1429 (**D**) and YTHDF1 (**L**) was not significantly different.

**Figure 13:**
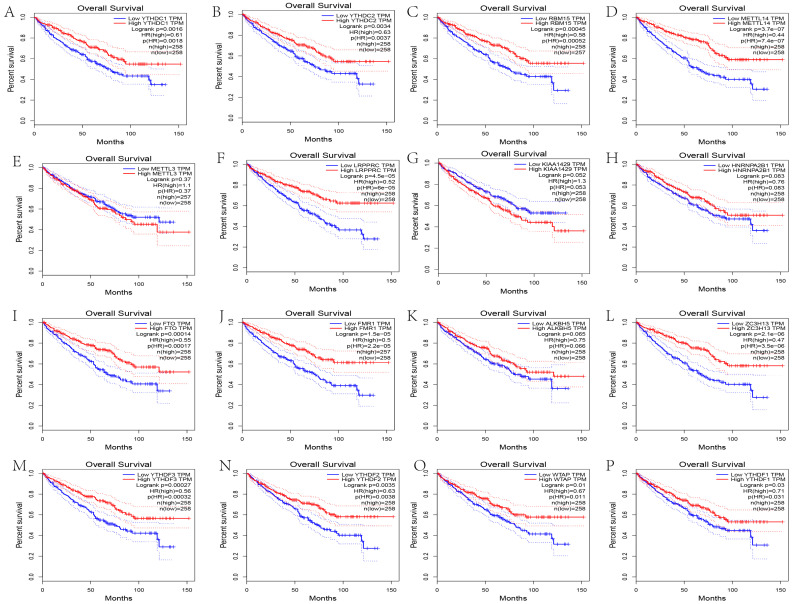
OS analysis of m6A-related genes using GEPIA. Kaplan–Meier plots demonstrate that high expression of YTHDC1 (**A**), YTHDC2 (**B**), RBM15 (**C**), METTL14 (**D**), METTL3 (**E**), LRPPRC (**F**), KIAA1429 (**G**); HNRNPA2B1 (**H**); FTO (**I**), FMR1 (**J**), ALKBH5 (**K**); ZC3H13 (**L**), YTHDF3 (**M**), YTHDF2 (**N**), WTAP (**O**), YTHDF1 (**P**) was associated with increased OS.

To understand the relationship between hub genes and m6A-related genes in RCC, we first analyzed the expression of m6A-related genes in tumor and normal tissues. The analysis indicated that FTO, ALKBH5, YTHDC2, WTAP, and RBM15 were highly expressed in the tumor group, and METTL14, METTL3, LRPPRC, HNRNPA2B1, FMR1, ZC3H13, YTHDF3, YTHDF2, and YTHDC1 were more highly expressed in normal tissues (Figure 11B and Figure 12). Kaplan–Meier plots of overall survival demonstrated that higher expression of YTHDC1, YTHDC2, RBM15, METTL14, LRPPRC, FTO, FMR1, ZC3H13, YTHDF3, YTHDF2, WTAP, and YTHDF1 was associated with increased OS (Figure 13). Then, the correlation between hub genes and m6A-related genes was determined.

Detailed information is shown in Table 2. The numbers represent the strength of the correlation, and red, blue, and gray colors indicate positive, negative, and nonsignificant correlations, respectively. Circos visualized the correlation, which showed that P2RY8, ERBB2, CAT, and TIMP1 had a close correlation with m6A-related genes (Figure 11A). Additionally, several high and very confidently predicted m6A sites were found on the mRNAs of the hub genes, and the specific sequence and location information are shown in Figure 11C. Furthermore, to understand the potential interactive pattern, we drew a predicted interactive diagram using data from the ENCORI and catPARID databases (Figure 11D). The results indicated that P2RY8, ERBB2, CAT, and TIMP1 may be methylated by METTL3 or METTL14 and then recognized by multiple RNA-binding proteins.

## 4. Discussion

Some RCC patients, especially advanced cases, usually do not have a good prognosis [23]. The lack of efficient molecular medication is one of the reasons hindering RCC treatment. Our study identified the potential biomarkers and therapeutic targets. The possible molecular mechanisms were exploited at the same time.

In our study, we screened 275 downregulated and 185 upregulated genes using 3 GEO datasets and the TCGA dataset. To choose robust biomarkers, protein–protein interaction (PPI) analysis was performed using the STRING web server. Additionally, the PPI network was further processed through the plugin MCODE and cytoHubba of Cytoscape. Eighty-two genes were screened out. To understand the potential mechanism, GO and KEGG enrichment analyses were carried out. The results illustrated that the top 3 biological process terms and the top 1 KEGG term were related to immunity. RCC is an immunogenic tumor [24]. Meanwhile, through cMAP analysis of 82 candidate hub genes, we found some antagonistic molecules that can be candidate drugs for the treatment of RCC. LASSO and random forest were employed together to select the most powerful variables for prognosis. Finally, six hub genes (ERBB2, CASR, P2RY8, CAT, PLAUR, and TIMP1) were identified. To further determine the predictive power and possible mechanisms of hub genes, multi-dimensional analysis was performed, including survival analysis, association with clinicopathological features, correlation analysis with m6A-related genes, and immune infiltration analysis.

Survival analysis demonstrated that low expression of TIMP1 and PLAUR and high expression of CASR, CAT, ERBB2, and P2RY8 were associated with a good prognosis, including OS and DFS. TIMP1 and PLAUR had a higher expression trend in the better subtype groups of clinicopathological features. CASR, CAT, ERBB2, and P2RY8 had a higher expression trend in the poor subtype groups of clinicopathological features. These analysis results suggest that the six hub genes play an important role in the progression of RCC. This consequence is partially consistent with previous studies, such as those involving ERBB2, which is rarely expressed in renal cell cancer and is involved in RCC oncogenesis [25]. CASR plays a vital role in the mechanism of bone metastasis in RCC [26]. TIMP1 indicates a poor prognosis of RCC and accelerates tumorigenesis [27]. However, P2RY8, PLAUR, and CAT have rarely been researched. In the present study, we found the expression of P2RY8 was interesting. It is expressed higher in tumors. However, with the clinical stage, histological grade, and the number of metastatic lymph nodes increasing, its expression decreased. This seems to be a paradox. In fact, a similar phenomenon does exist in other studies. For example, obesity is a clear risk factor for RCC; however, obesity can also improve the prognosis of RCC [28].

m6A occurs in the sixth-N-position of adenosine, which is the most abundant, prevalent, and conserved RNA modification in higher eukaryotes. Accumulating studies have indicated that m6A modification regulates tumor pathogenesis and progression in ccRCC. For example, METTL3 had a higher expression in ccRCC tissues and enhanced the cell viability, migration, and invasion abilities via regulating m6A modification of HHLA2 [29]. FTO inhibited autophagy of ccRCC cells and promoted tumor progression through an m6A-IGF2BP2-dependent mechanism, and SIK2 was their m6A target [30]. On the other hand, YTHDF2 recognized METTL14-mediated m6A markers on NEAT1_1 to accelerate the degradation of NEAT1_1, thus inhibiting the proliferation and migration ability of RCC cells [31].

To determine whether the mechanism of the six hub genes in RCC is related to m6A modification, a detailed analysis was performed. There are many m6A-related genes, and we chose 16 representative genes based on the literature [17]. According to our research, five m6A-related genes were highly expressed in the tumor group, and nine m6A-related enzymes had higher expression in normal tissues. High expression of twelve m6A-related genes is associated with increased OS. These results are consistent with a previous study [32]: Jiawu Wang indicated most of the m6A regulators have different expressions in RCC tissue, and METTL14 and METTL3 were identified as two powerful independent prognostic regulators after further analysis. Their results illustrate that m6A modification does play an important role in RCC. In our study, we found the expression of P2RY8, ERBB2, CAT, and TIMP1 had a close correlation with the expression of most m6A regulators. Additionally, several high and very confidently predicted m6A sites were found on the mRNAs of the hub genes. Finally, we drew a possible interactive diagram according to the data from ENCORI and catRAPID. These results all strongly support that ERBB2, CAT, P2RY8, and TIMP1 can regulate the development of RCC in an m6A-dependent manner. By searching the PubMed website, we found some similar m6A studies on these four hub genes. In esophageal squamous cell carcinoma, ERBB2 was identified as the target of FTO using m6A-seq and RNA-seq assays, and YTHDF1 then binds and stabilizes ERBB2 mRNA. Finally, the expression of ERBB2 increases and promotes cancer [33]. TIMP1 is more highly expressed in high-grade glioma and highly correlates with m6A markers in glioma, and TIMP1 serves as a potential biomarker for prognosis [34]. However, there are few RCC-related studies. Regarding P2RY8 and CAT, we found few m6A-relevant studies. However, we must explain as follows: 1. These results only reveal underlying m6A mechanisms with hub genes in ccRCC. In our research, one hub gene can simultaneously promote/inhibit the expression of methylase and demethylase. However, we cannot simply conclude they increase/decrease the m6A level of ccRCC or promote/inhibit tumor progression because one methylase can modify oncogenes and anti-oncogenes to play a synergistic role. Additionally, one m6A regulator can promote proliferation and inhibit invasion and migration, which seem to be a reverse phenotype in a tumor [35]. In addition, the m6A gene can act as an independent prognostic factor rather than an m6A regulator [36]. 2. Although m6Asites localize primarily around 5′ and 3′UTR [37], the study also indicates about 35% m6A site in coding regions (CDS) [38]. These results are consistent with our findings. It is worth mentioning that CDS m6A also play an important regulatory role. For example, it can disrupt tRNA selection and slow down translation elongation [39]. Additionally, research showed it induces ribosome pausing in a codon-specific way; removing CDS m6A leads to a further decrease in translation unexpectedly [38].

Tumor-infiltrating immune cells (TIICs) in the tumor microenvironment greatly determine tumor progression; they are promising targets for drug therapy and have demonstrated potential therapeutic value [8]. Our study indicated that TIICs are differently distributed in different subgroups of clinicopathological features. The data showed that M2 macrophages and CD8 T cells obviously infiltrated the tumor group, and this phenomenon is consistent with a previous study that showed that RCC is mainly infiltrated by T cells and myeloid cells [24]. Interestingly, recent research shows that M2 macrophages are heavily enriched in advanced RCC [22]. However, our study indicated that a positive clinicopathological feature group had a higher infiltration of M2 macrophages. This inconformity may be related to the different methods of measurement. The study above applied single-cell RNA sequencing, and our data used ordinary RNA sequencing. On the other hand, although the enrichment of M2 macrophages decreased in the advanced groups, we did not determine their functional state. Finally, studies found that CD8+ T cells and macrophages can interact to induce immune dysfunction [22], and we also did not obtain any data about whether they interacted and the intensity of this interaction. These reasons above may lead to different clinical phenotypes. In addition, specific markers of immune cells can be expressed in a proportion of cancer cells. This immune-like transcriptional reprogramming is considered to be an important hallmark of cancer [40]. Additionally, this immune mimicry may have an impact on the results of immune infiltration.

Our study also showed that regulatory T cells, resting mast cells, and resting dendritic cells are predictors for prognosis in RCC. Meanwhile, the expression of PLAUR, CAT, P2RY8, ERBB2, and TIMP1 is close to the infiltration of TIICs. This finding reveals that the five hub genes above may regulate tumor progression in an immunity-dependent pattern and can be potential biomarkers for immunotherapy.

This research has some limitations. The main issue is a bioinformatics design without experimental verification. Additionally, the analysis of potential mechanisms is superficial, and further precise exploration should be carried out, such as the expression of hub genes at the post-transcriptional level, precising m6A sites of hub genes. Moreover, we did not obtain the data on which patients underwent immune therapy and did not explore the relationship between hub gene expression and immune response.

## 5. Conclusions

We identified six hub genes through multi-dimensional screening. They all possess strong predictive value for prognosis and clinicopathological features. Meanwhile, we found that m6A modification and immune infiltration may be involved in the development of RCC. Furthermore, hub genes may regulate the progression of RCC in an m6A- and immunity-dependent manner. Overall, our study provides new biomarkers for treatment and diagnosis.

## Figures and Tables

**Table 1:** The predicted antagonistic molecules for RCC using the cMAP database. The antagonistic molecules were screened for RCC by cMAP analysis of 82 candidate hub genes. The top 10 ranked molecules are listed in this table. These molecules can induce opposite disturbances in RCC. Thus, these molecules can be candidate drugs for the treatment of RCC.

The Predicted Antagonistic Molecules for RCC Using the cMAP Database			
Name	Target	Moa	Raw-Score
miglustat	UGCG|GAA|SLC5A4	Glycosylation inhibitor	0.7831
torasemide	SLC12A1|CYP2C8|SLC12A2	Thromboxane receptor antagonist	0.7433
AZ-628	BRAF|RAF1	RAF inhibitor	0.7421
atracurium	CHRNA2	Acetylcholine receptor antagonist	0.733
PA-824	FASN	Nitric oxide donor	0.729
prochlorperazine	DRD2|DRD1|DRD3|DRD4	Dopamine receptor antagonist	0.7268
wiskostatin	WAS|WASL	Neural Wiskott–Aldrich syndrome protein inhibitor	0.722
carbamazepine	SCN1A|SCN3A|SCN5A|ABCB1|CYP1A2|CYP3A4|SCN10A|SCN11A|SCN2A|SCN4A|SCN7A|SCN8A|SCN9A	Carboxamide antiepileptic	0.721
CCT-031374	CTNNB1	WNT inhibitor	0.7207
LFM-A13	BTK	BTK inhibitor	0.7199

**Table 2:** Correlation analysis between hub genes and m6A-related genes. Correlation analysis was performed using the TIMER2.0 website. The numbers represent the strength of the correlation. Red indicates a positive correlation. Blue indicates a negative correlation. Gray color means nonsignificant. The expression of CASR, CAT, ERBB2, and P2RY8 is positively related to the expression of m6A-related genes. The expression of TIMP1 is negatively related to the expression of m6A-related genes.

Correlation Analysis between Hub Genes and m6A Related Genes						
	CASR	CAT	ERBB2	P2RY8	PLAUR	TIMP1
ALKBH5	0.246	0.38	0.436	0.384	0.002	−0.187
FTO	0.134	0.348	0.261	0.415	0.024	−0.192
HNRNPA2B1	0.162	0.159	0.203	0.328	0.081	−0.173
KIAA1429	0.134	0.298	0.216	0.434	0.058	−0.137
METTL14	0.278	0.574	0.454	0.538	−0.145	−0.417
METTL3	0.193	0.119	0.217	0.265	−0.053	−0.236
RBM15	0.244	0.309	0.293	0.515	0.095	−0.232
WTAP	0.12	0.261	0.062	0.43	0.126	−0.131
YTHDC1	0.276	0.431	0.528	0.601	−0.119	−0.342
YTHDC2	0.153	0.397	0.22	0.418	−0.027	−0.3
YTHDF1	0.222	0.336	0.395	0.32	0.019	−0.197
YTHDF2	0.252	0.41	0.3	0.542	0.013	−0.182
YTHDF3	0.245	0.512	0.228	0.327	−0.029	−0.313
ZC3H13	0.256	0.426	0.418	0.578	−0.041	−0.27
FMR1	0.228	0.413	0.362	0.319	−0.065	−0.304
LRPPRC	0.182	0.532	0.412	0.358	−0.192	−0.455
spearman’s *p*:	positive correlation (*p* < 0.05)					
spearman’s *p*:	positive correlation (*p* < 0.05)					
spearman’s *p*:	positive correlation (*p* > 0.05)					

## Data Availability

The row data included in this study are available in TCGA (https://portal.gdc.cancer.gov/, (accessed on 19 March 2022)) and GEO (https://www.ncbi.nlm.nih.gov/geo/, (accessed on 16 March 2021)).

## Supplementary Materials

The following are available online at https://www.mdpi.com/article/10.3390/genes13112059/s1, Table S1: The genes list of “Gene list1”, ”Gene list2”, ”Candidate hub genes”, ”Gene list3”, ”Gene list4”; Table S2: The summary of 6 hub genes using GeneCards (https://www.genecards.org/). Degree of location confidence ranging from 1 to 5 (highest).
