# Supplementary material for: Identifying Prognostic Biomarkers Related to m6A Modification and Immune Infiltration in Renal Cell Carcinoma
Source: Genes (Basel). 2022 Nov 7;13(11):2059. doi: 10.3390/genes13112059 (PMC9690957; doi:10.3390/genes13112059)
Supplement: Supplementary file 1 [file genes-13-02059-s001.zip › genes-1965968-supplementary.pdf]

Supplementary Table S1:

The genes list of “Gene list1”, ”Gene list2”, ”Candidate hub genes”, ”Gene list3”, ”Gene list4”.

Gene list1 (144 genes)	Gene list2 (100 genes)	candidate hub genes (82 genes)	Gene list3 (18 genes)	Gene list4 (6 genes)
AOAH	ITGB2	AOAH	PLG	ERBB2
ARHGDIB	TYROBP	ARHGDIB	P2RY8	CASR
CASP1	FCGR3A	CASP1	TIMP1	P2RY8
CCL5	ITGAM	CCL5	PLAUR	CAT
CD163	CSF1R	CD163	CAT	PLAUR
CD27	C1QA	CD27	GIMAP4	TIMP1
CD53	C1QB	CD53	ERBB2	
CD8A	TLR7	CD8A	PARVG	
CORO1A	LY86	CORO1A	CASR	
CXCL10	LCP2	CXCL10	ARG2	
CXCL9	PLEK	CXCL9	CSF3R	
FCGR2A	FCGR2A	FCGR2A	ITGA4	
FCGR3A	IL10RA	FCGR3A	TLR2	
GIMAP4	TLR2	GIMAP4	CD14	
GZMK	CD163	GZMK	RGS1	
IGSF6	LAPTM5	IGSF6	TLR7	
IL2RB	CD53	IL2RB	TGFB1	
ITGAM	C1QC	ITGAM	FCGR2A	
ITGB2	CD14	ITGB2		
LAPTM5	DOCK2	LAPTM5		
LCP1	HCLS1	LCP1		
LCP2	PARVG	LCP2		
MS4A4A	FERMT3	MS4A4A		
MS4A6A	CD8A	MS4A6A		
NCF2	CCL5	NCF2		
NKG7	CXCL9	NKG7		
PLEK	CXCL10	PLEK		
RGS1	IGSF6	RGS1		
SLAMF8	MNDA	SLAMF8		
THEMIS2	ITGAL	THEMIS2		
TLR2	VAV1	TLR2		
TYROBP	CORO1A	TYROBP		
ALB	CD300A	ALB		
APBB1IP	NCKAP1L	APBB1IP		
C1QA	APBB1IP	C1QA		
C1QB	EVI2B	C1QB		
C1QC	ALB	C1QC		

CD14	CLEC7A	CD14
CD300A	TREM2	CD300A
CLEC7A	IL2RG	CLEC7A
CSF1R	MS4A6A	CSF1R
DOCK2	CYTH4	DOCK2
EVI2B	NCF2	EVI2B
FERMT3	ARHGDIB	FERMT3
HCLS1	CASP1	HCLS1
IL10RA	GIMAP4	IL10RA
LY86	RGS1	LY86
MNDA	SLAMF8	MNDA
NCKAP1L	LCP1	NCKAP1L
PARVG	IL2RB	PARVG
TLR7	CD27	TLR7
TREM2	NKG7	TREM2
VAV1	THEMIS2	VAV1
ACADM	MS4A4A	KCNJ1
ACAT1	FN1	SLC12A3
ALDH6A1	MYO1F	ERBB2
ALDOB	GZMK	ANGPT2
HADH	C1orf162	AQP2
LDHB	EGF	BSND
PCCA	AOAH	CASR
CLDN16	TGFB1	CD3D
KCNJ1	TIMP1	CLCNKB
NPHS2	IDO1	CSF3R
RHCG	INPP5D	EGF
SLC12A3	ARG2	FN1
SLC34A1	RASAL3	INPP5D
UMOD	CD3D	ITGA4
ERBB2	PTPN22	ITK
NRP2	PLG	P2RY8
PLXNB1	CSF3R	PLG
PLXNC1	PLAUR	PTPN22
PLXND1	PLAT	SCNN1A
SEMA3B	ITGA4	SELPLG
SEMA6D	P2RY8	WNK4
ANGPT2	SELPLG	ARG2
AQP2	KNG1	CAT
BSND	CD3G	IDO1
CASR	CAT	KNG1
CD3D	GATA3	PLAT
CLCN5	KCNJ1	PLAUR
CLCNKA	BSND	TGFB1

CLCNKB	GPR174	TIMP1
CSF3R	HLA-DPA1	
EGF	ITK	
FGF1	FPR3	
FN1	GPR34	
HRG	AQP2	
INPP5D	WNK4	
ITGA4	SCNN1A	
ITK	ERBB2	
NR3C2	SLC12A1	
P2RY8	SLC12A3	
PAG1	CLCNKB	
PDGFRA	CST7	
PLG	NEDD4L	
PTPN22	SLAMF6	
SCNN1A	TNFRSF9	
SELPLG	CASR	
WNK4	SIGLEC9	
ACADSB	ANGPT2	
BCKDHB		
DBT		
HMGCS2		
PCK1		
PDHB		
SCD		
SLC25A4		
SLC25A5		
SUCLG1		
UQCRFS1		
TMEM116		
TMEM30B		
TMEM61		
TMEM72		
ANLN		
CDCA2		
NUSAP1		
TPX2		
CA9		
EPCAM		
GPC3		
KRT7		
ARG2		
CAT		
IDO1		

KNG1
PLAT
PLAUR
TGFB1
TIMP1
ABAT
HIGD1A
NDUFA4
OGDHL
PEPD
SDHD
SOD3
UQCRC1
CALB1
PVALB
SLC1A3
APOBEC3G
SAMHD1
SLFN11

Supplementary Table S2:

The summary of 6 hub genes using GeneCards (https://www.genecards.org/). Degree of location confidence ranging from 1 to 5(highest).

Gene symbol	Desription	Gene category	Location	Location confidence	Summary of function
CAT	Catalase	protein coding	cytosol/peroxisome/mitochondrion/extracellular	5/5/4/4	Diseases associated with CAT include Acatalasemia and Peroxisome Biogenesis Disorder 1B. Among its related pathways are Glucose / Energy Metabolism and Detoxification of Reactive Oxygen Species. Gene Ontology (GO) annotations related to this gene include protein homodimerization activity and enzyme binding.

ERBB2	Erb-B2 Receptor Tyrosine Kinase 2	protein coding	cytosol/endosome/nucleus/plasma membrane/extracellular	5/5/5/4	Diseases associated with ERBB2 include Visceral Neuropathy, Familial, 2, Autosomal Recessive and Glioma Susceptibility 1. Among its related pathways are Hepatocyte growth factor receptor signaling and PI5P, PP2A and IER3 Regulate PI3K/AKT Signaling. Gene Ontology (GO) annotations related to this gene include identical protein binding and protein kinase activity. An important paralog of this gene is EGFR.
P2RY8	P2Y Receptor Family Member 8	protein coding	plasma membrane	5	Diseases associated with P2RY8 include B-Lymphoblastic Leukemia/Lymphoma With Iamp21 and Intellectual Developmental Disorder, Autosomal Dominant 33. Among its related pathways are Purinergic signaling. Gene Ontology (GO) annotations related to this gene include G protein-coupled receptor activity and G protein-coupled purinergic nucleotide receptor activity. An important paralog of this gene is F2R.
PLAUR	Plasminogen Activator, Urokinase Receptor	protein coding	extracellular/plasma membrane/endoplasmic reticulum	5/5/4	Diseases associated with PLAUR include Ureter, Cancer Of and Focal Segmental Glomerulosclerosis. Among its related pathways are Apoptotic Pathways in Synovial Fibroblasts and Response to elevated platelet cytosolic Ca2+. Gene Ontology (GO) annotations related to this gene include signaling receptor binding and signaling receptor activity. An important paralog of this gene is LYPD3.
TIMP1	TIMP Metallopeptidase Inhibitor 1	protein coding	extracellular/endoplasmic reticulum	5/4	Diseases associated with TIMP1 include Oral Submucous Fibrosis and Gingival Overgrowth. Among its related pathways are Apoptotic Pathways in Synovial Fibroblasts and Extracellular matrix organization. Gene Ontology (GO) annotations related to this gene include cytokine activity and protease binding. An important paralog of this gene is TIMP2.
